# Gender disparity in epidemiological trend of HIV/AIDS infection and treatment in Ethiopia

**DOI:** 10.1186/s13690-018-0299-8

**Published:** 2018-09-17

**Authors:** Tadele Girum, Abebaw Wasie, Kifle Lentiro, Ebrahim Muktar, Teha Shumbej, Mesfin Difer, Mulugeta Shegaze, Abdulsemed Worku

**Affiliations:** 1 Department of Public Health, College of Medicine and Health Sciences, Wolkite University, Wolkite, Ethiopia; 2 Department of Medical Laboratory Science, College of Medicine and Health Sciences, Wolkite University, Wolkite, Ethiopia; 3 Department of Midwifery, College of Medicine and Health Sciences, Wolkite University, Wolkite, Ethiopia; 4 Department of Public Health, College of Medicine and Health Sciences, Arba Minch University, Arba Minch, Ethiopia; 5 Department of Medicine, College of Medicine and Health Sciences, Wolkite University, Wolkite, Ethiopia

**Keywords:** Gender disparity, HIV/AIDS and gender, Antiretroviral therapy, Trend of HIV

## Abstract

**Background:**

The HIV/AIDS epidemic has been fuelled by gender inequality and disparity resulted in violation of women reproductive right. The “feminization” of the pandemic is more apparent in Sub-Saharan Africa where the larger numbers of HIV infected people are living. Although they carry the higher proportion of HIV cases; access to care and treatment is lower among women. In Ethiopia where HIV is prevalent and gender violence is common, the disparity may be higher. Therefore, this research aimed to assesses trends in gender disparity in the HIV/AIDS epidemic in Ethiopia to bring evidence for action.

**Methods:**

This study was conducted using aggregates of HIV/AIDS indicator data from 1990 to 2016 of UNAIDS data bases. The data was compiled and analyzed with excel and STATA Version 11. The trend was assessed, gender difference was measured and rate of change was compared between genders and specific age groups.

**Result:**

Adult females (age 15+) accounted 61.5% of the HIV cases and new infection among adults. While, adolescent females (age 10-19) and young women (age 15-24) accounted 52.3 and 57.5% of prevalent cases and 74 and 68% of new infection in their age category respectively. HIV is 1.62 times more prevalent among adult women than men. Since 1990, HIV cases among adults has risen markedly in the first decade with 24 and 20%, then declined by 41.5% in the second decade and rose again by 5 and 8.7% among women and men respectively. The overall prevalence is declined by 72.4 and 71.5% from the maximum record. Women and men have equal access for ART; 62% of men and 61% of women from all adults living with HIV were on ART. While 61% of deaths were among adult women and the death rate is similar among adolescent women and men. AIDS- related death has been declined by 76% from the maximum record.

**Conclusion:**

HIV/AIDS prevalence, new infection and AIDS-related death are by far higher among adult women than men. While the coverage of treatment and HIV care is equal among both genders. Vulnerable age groups (adolescent females and young women) take the lion’s share of the new infections and prevalent cases. Therefore due attention is needed to avert gender disparity with a particular emphasis for adolescents and young women.

**Electronic supplementary material:**

The online version of this article (10.1186/s13690-018-0299-8) contains supplementary material, which is available to authorized users.

## Background

Human immunodeficiency virus (HIV) infection remains the leading cause of morbidity and mortality throughout the world [1]. The epidemic has been fuelled by gender inequality and disparity, which resulted in violation of women reproductive right [1, 2]. Unequal power relations, sexual coercion and violence are a widespread phenomenon faced by women of all age groups that have an array of negative effects on female sexual, physical and mental health which predispose to HIV infection. HIV/AIDS infection reveals the disastrous effects of discrimination against women on human health and on the socio-economic structure of the society [1, 3].

Globally, by the end of 2016, an estimated 36.7 million people were living with HIV and of these, as many as 17.8 million (51.6% of adults) were women [1, 4]. Differences in the number of new HIV infection between men and women are more pronounced at younger ages where, new infection among young women (aged 15–24 years) were 44% higher than they were among men in the same age group [2, 4]. Of the 1.8 million new HIV infections and 1 million AIDS-related death by the year women accounted the larger share as well [1, 4].

The “feminization” of HIV pandemic is more apparent in Sub-Saharan Africa; the region where 76% of the total HIV infected people, 76% of the total new HIV infections, and 75% of the total HIV/AIDS related deaths were recorded in 2015 [1, 2]. In such high-prevalence settings, women remain at unacceptably higher risk of HIV infection. Women account for 56% of the total number (6.1 million) of people living with HIV in the western and central Africa region and 59% of the 19.4 million people living with HIV in eastern and southern Africa region [1, 3, 4].

In Ethiopia, where HIV is characterized by a low-intensity mixed epidemic and self-sustaining transmission, women account for 433,763 of approximately 718,500 estimated cases of people living with HIV in 2016 (constituting 60% of all HIV infections) [5]. Similarly, 58% of new infections and 57% of AIDS-related death was among females [5]. It is also evidenced that women are carrying higher burden of the disease [1, 5–9]. In 2003, Ethiopia has introduced its ART program and in 2005 free ART service was launched in accordance with the world health organizations (WHO) guide line [5, 6].

Some researchers reported that HIV transmission through the two most common routes (sexual and blood transfusion) as well as its prevalence among women is higher than men [2, 3]. Their exposure to the virus at an earlier age coupled with physiological factors increases their risk of infection through sexual route [1–4]. Similarly, exposure to blood transfusion due to anemia and complications during pregnancy and childbirth along with low access to information and treatment for other infections which facilitate the transmission of HIV [1, 3] and onset of AIDS, including sexually transmitted infections put women to higher risk of HIV infection [9–13].

Also they account higher proportion of HIV cases [1, 4]. Meanwhile, access to care and treatment is lower among women [2, 4]. The social and familial burden they have along with ethnic and gender disparities that are common in most developing countries limit women from accessing HIV care and treatment [4, 10, 13]. Thus, the difference in treatment access, time of presentation, biological difference in disease progression and immunological response among women and men may impose a difference in progression of the disease and women may carry the highest risk of AIDS-related mortality [9–13].

Of course, AIDS-related illnesses remain the leading cause of death among women of reproductive age (15–49 years) globally [1], and they are the second leading cause of death for young women aged 15–24 years in Africa [2, 3]; the impact of HIV and AIDS reaches far beyond the health sector with severe economic and social consequences and the impact is much more severe on women than on men [1, 9]. Women (age 15+) and girls (females younger than 15 years) seems to bear the impact of the pandemic in many ways and the disease also disproportionately affects them psychologically, socially and economically [10, 11].

Ethiopia is among the Sub-Saharan countries where HIV is prevalent and gender violence is common which may results in gender disparity in HIV/AIDS prevalence, incidence and treatment [3, 7, 9]. To the extent of our search the level of gender disparity in HIV/AIDS epidemiology was not well understood. Therefore, this research aimed to assesses gender disparity in epidemiological trend of HIV/AIDS epidemics in Ethiopia; which have greater importance to bring special evidence that could help planners, implementers and aid organizations in the country and abroad to take evidence-based action.

## Methods

### Study design, settings and population

This research used data from international data bases (USAIDS, WHO and World Bank) to assess trends in gender disparity in HIV/AIDS in Ethiopia. Ethiopia is the second most populous country in Africa next to Nigeria, with a population estimated at 99,390, 000 in 2015 of which 83.86% live in rural areas and 51% are females [6, 14]. While HIV/AIDS in Ethiopia has been recognized since the mid-1980, the epidemic has remained a major public health problem, largely affecting people of productive and reproductive age, in particularly women. To overcome this problem, a larger preventive and curative activity has been undertaken. Regular monitoring and evaluation programs through surveillance and survey are taking place, which are the basic data sources of this research [6, 14].

### Study variables, sources of data and data collection procedure

Specialized agencies of the United Nation including WHO, UNAIDS, UNDP and other intergovernmental organizations like the World Bank compile data of each member countries for policy, research and other purposes. The major sources of HIV/AIDS related data is achieved in UNAIDS (http://aidsinfo.unaids.org/) data base which also served as the major sources of information for this research. Data related to HIV/AIDS incidence, prevalence, test, treatment, prevention and outcomes are available by regional, sub-regional and national level as well as stratified by age and sex. Date bases of other agencies of United Nation, WHO (http://www.who.int/healthinfo/statistics/en/), UNICEF, UNDP and World Bank (https://data.worldbank.org/) were used as additional source and for verification purposes. In addition to theses source of raw data, published form of data was used from WHO and the Ethiopian ministry of health data bases are used to strengthen the discussion.

Majority of the data sources were validated by the agency, while some are direct reports submitted from each country which is compiled through available national surveillance, national surveys, censuses, vital statistics, modeled prediction (forecasts) and large surveys/researches. Country-specific estimates were based on the available interagency estimates/data available since 1990 for HIV prevalence, incidence and AIDS related death and back to 2010 for ART/number of people enrolled and coverage.

The study indicators were selected purposively from UNAIDS Global AIDS Monitoring 2017 guide line [15]. The one which are proxy measures of the HIV/AIDS burden (HIV prevalence and incidence, ART enrolment and coverage, AIDS related death) and important for comparison purpose and the one which have available data are selected. Finally, indicators like: Percentage and/ or number of People living with HIV, People living with HIV who are on antiretroviral therapy and AIDS-related mortality are used for assessing the trend of HIV and to compare gender difference through the course of the epidemics.

The data was extracted by the investigators and their assistants who are experienced in data mining. By using preformed document extraction check list data from each preselected indicator was extracted from the identified data base. An excel sheet to which the values of study indicators were filled was used to collect the relevant indicators of the study across the study duration. All indicators were defined according to their standard definition given by the source of the indicators at UNAIDS and WHO.

### Operational definition

In accordance to the UNAIDS and World Health Organization’s definition, the following terms were adopted [15].

- **People living with HIV who know their HIV status**: Percentage of people living with HIV who know their HIV status at the end of the reporting period (among adults)
- **People living with HIV on antiretroviral therapy**: Percentage and number of adults on antiretroviral therapy among all adults living with HIV at the end of the reporting period
- **Retention on antiretroviral therapy at 12 months**: Percentage of adults and children living with HIV known to be on antiretroviral therapy 12 months after starting
- **People living with HIV who have suppressed viral loads**: Number and percentage of people living with HIV who have suppressed viral loads at the end of the reporting period with the last or recent viral load measurement.
- **AIDS mortality**: Total number of people who have died from AIDS-related causes per 100 0 population (among adults, adolescent and young ages).
- **Mother-to-child transmission of HIV**: Estimated percentage of children newly infected with HIV from mother-to-child transmission among total children of HIV infected mother delivered in the past 12 months
- **Preventing the mother-to-child transmission of HIV**: Percentage of pregnant women living with HIV who received antiretroviral medicine to reduce the risk of mother-to-child transmission of HIV
- **HIV incidence**: Number of people newly infected with HIV in the reporting period per 1000 uninfected population
- **Girls**: is used in this paper to express females aged lower than 15 years
- **Women**: is used in this paper to express females aged 15 and above.
- **Female**: is used in this paper to express biological difference to male at any age.
- **Young age:** are a segment of population with age category between 15 and 24
- **Adolescents:** are a segment of population with age category between 10 and 19
- **Adults**: are a segment of population with age 15 years and above

### Statistical analysis

After the data was obtained from different sources, it was compiled with excel, each variable was checked for completeness and consistency. Whenever a series of data was obtained from more than one source validation was done and either UNAIDS or WHO’s data was used. Data was cleaned, coded and exported to STATA version 11 for Windows, and then exploratory data analysis carried out. The patterns of each selected indicators and their change were described numerically and graphically with line graphs that plotted using points on the X-Y axis, where X is the time in year (mostly from 1990 to 2016) and Y is value of the selected indicator for each year in number or percent. Changes were estimated and values were compared between male and female for all adults and specific age groups of adolescent and young ages which have public health importance. The significance of the difference between the two genders was determined through their confidence interval; whenever the confidence interval is non-overlapping it is considered as significantly different. The findings were compared and discussed in accordance with previous literatures and available standards.

## Results

### Gender disparity in HIV/AIDS infection

**Fig. 1:**
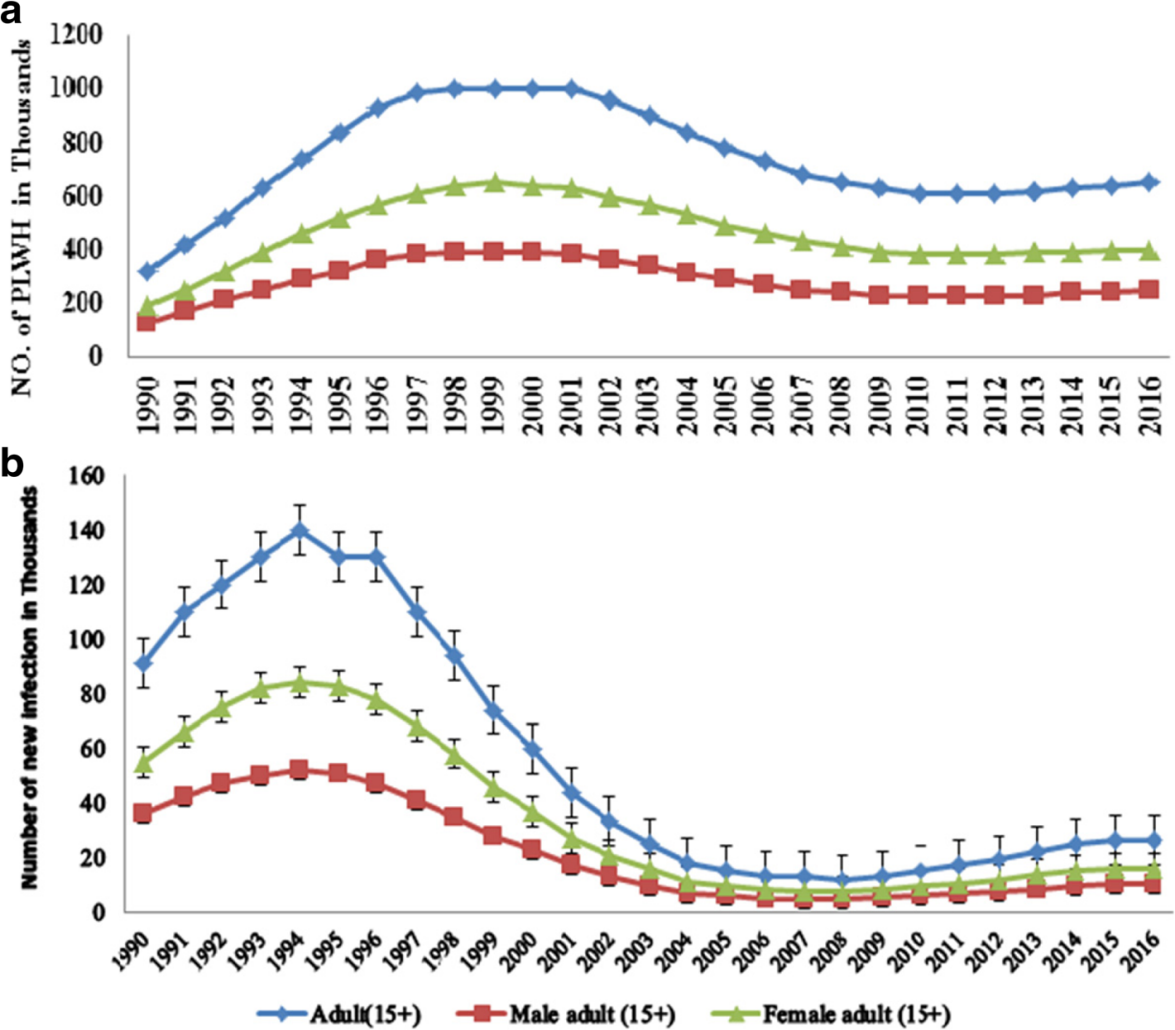
Number of HIV prevalent (**a**) and incident (**b**) cases by sex among adults of Ethiopia, 1990–2016

**Fig. 2:**
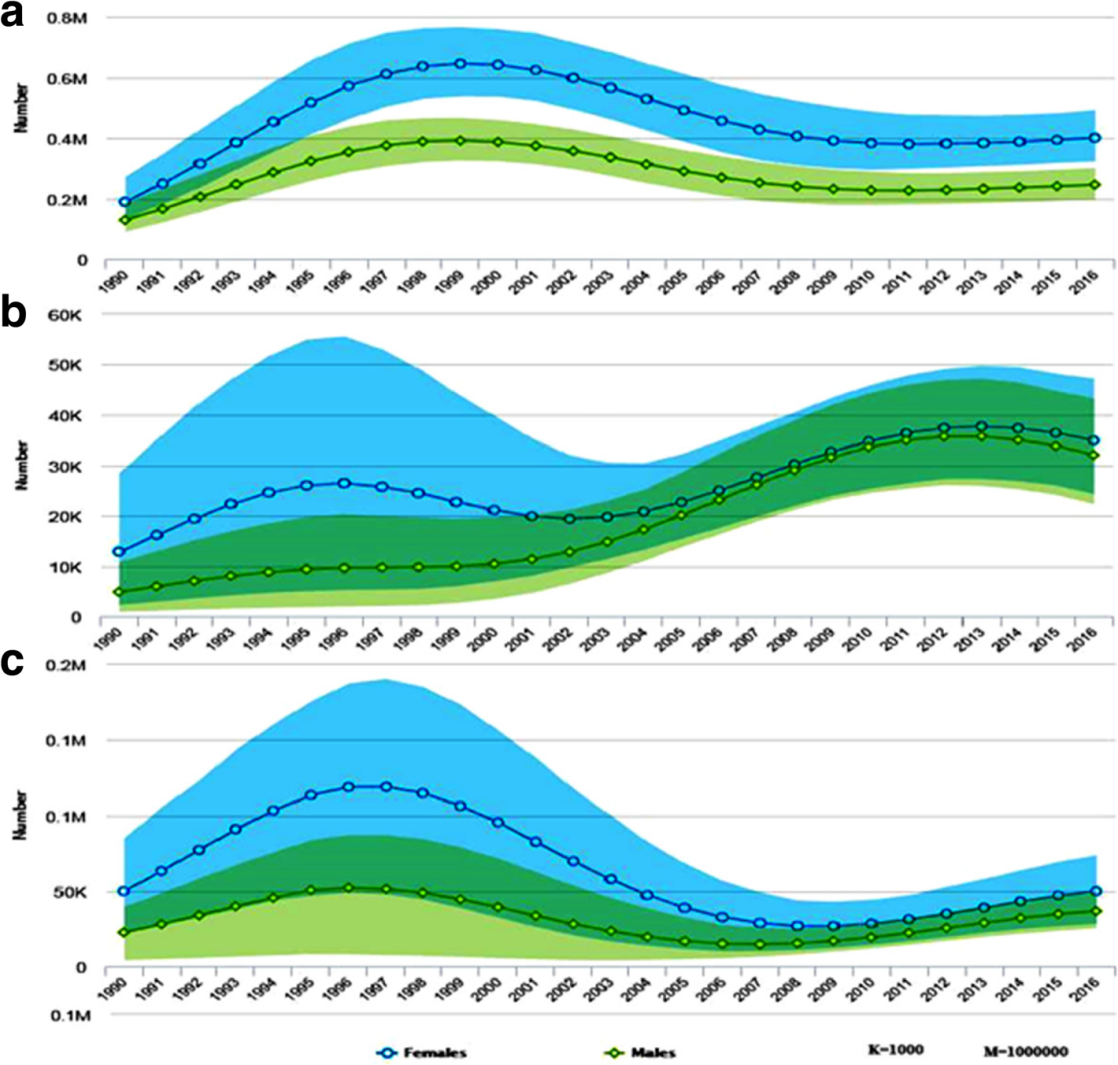
Number of HIV prevalent cases by age and sex among adults (**a**), adolescents (**b**) and young ages (**c**) of Ethiopia, 1990–2016

**Fig. 3:**
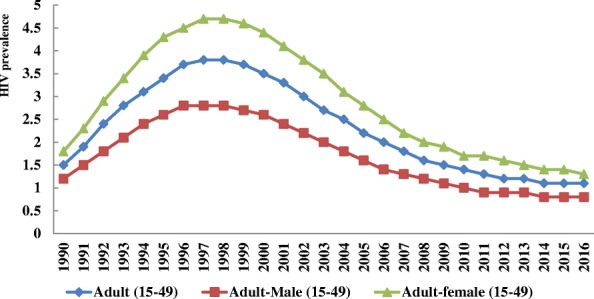
Prevalence of HIV infection by sex among adults of Ethiopia, 1990–2016

In 2016, there were 710,000 (570,000- 880,000) people living with HIV among the general population. Of which, 91.5% (650,000 cases) were among adults aged 15 and above years. Adult females accounted 400,000 (61.5%) of the 650,000 adult HIV cases. While, adolescent females (age 10-19) and young women (age 15-24) accounted 52.3% of the 67,000 and 57.5% of the 87,000 cases among adolescents and young age groups respectively. The overall prevalence rate of HIV/AIDS is 1.1% (0.8-1.3%) among all adults age 15-49, with a prevalence of 0.8% (0.6-1%) among men and 1.3% (1-1.7%) among women of the same age (Figs. 1, 2 and 3).

**Fig. 4:**
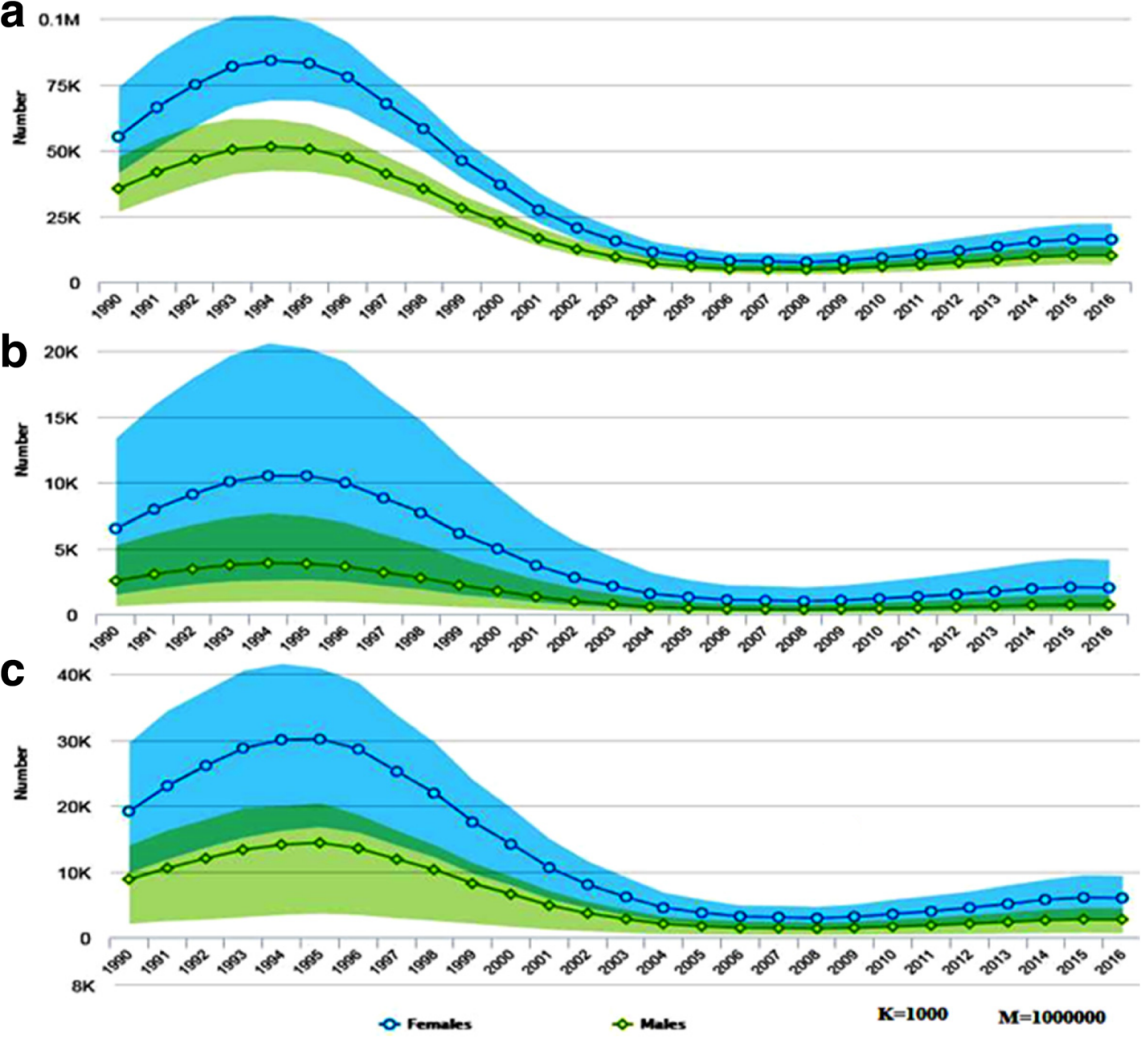
Number of new HIV infections by age and sex among adults (**a**), adolescents (**b**) and young ages (**c**) of Ethiopia, 1990–2016

In 2016, there were 26,000 (17,000–36,000) new infections among adults (age 15+). Adult Women disproportionately accounted 61.5% of the incidence in the age group. Similarly, 2000 of the 2700(1000–5600) new cases among adolescents and 6000 of the 8700 new cases among young people were women. i.e. women accounted 74% of new infection among adolescents and 68% of new infection recorded among young age people (Figs. 1 and 4).

Since 1990, the annual number of HIV cases (total prevalent cases) among adults (age 15+) has risen markedly in the first decade. During the time the number of HIV cases increased by 24 and 20% per year among women and male adults respectively. In the second decade, where HIV cases started to decline markedly, a 41.5% reduction in female cases from a peak level of 650,000 cases in 1999 to lowest level of 380,000 in 2010 and a 41% reduction in male cases from a peak level of 390,000 to 230,000 in the same year were observed. The prevalence of HIV was declined by 72.4 and 71.5% from the peak level of 4.7 and 2.8% in 1998 among women and men respectively (Figs. 1 and 3).

However, after remarkable decline for decades, since 2010 the number of HIV cases started to rise again by 5% among adult women (age 15+) and 8.7% among adult men (age 15+). The number of new infection (number of incident cases) also doubled within 8 years from the least record of 7500 cases in 2008 to 16,000 in 2016 and from 4700 to 10,000 by the same year in women and men respectively Additional file 1.

### Gender disparity in HIV/AIDS treatment and care

**Fig. 5:**
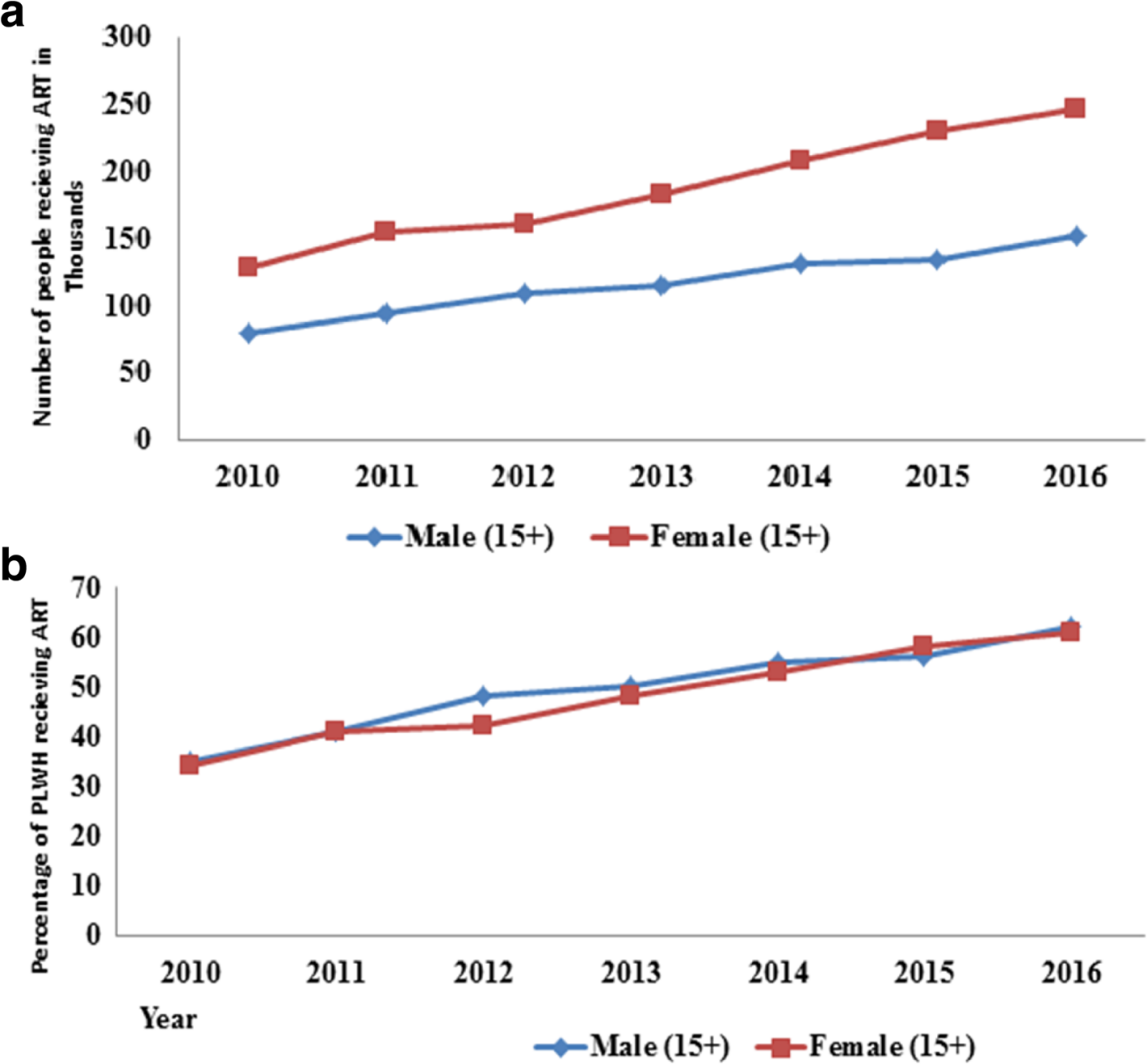
People living with HIV receiving ART: number of people receiving ART (**a**) and Coverage of ART (**b**) by sex among adults of Ethiopia, 2010–2016

Nationally, 420,000 clients are on ART; which means 59% of all people living with HIV or 88% of people living with HIV who know their HIV status are on treatment. Of these, 399,000 are adults (age 15+). That means, 152,000 of the 245,161 (62%) men and 247,000 of the 404,918 (61%) women from all adults living with HIV were on ART in 2016. Since 2010, the coverage of antiretroviral treatment has significantly increased by 13% per year with a 79% increment from the base line coverage of 34% among women to 61% (Fig. 5).

### Gender disparity in AIDS-related mortality

**Fig. 6:**
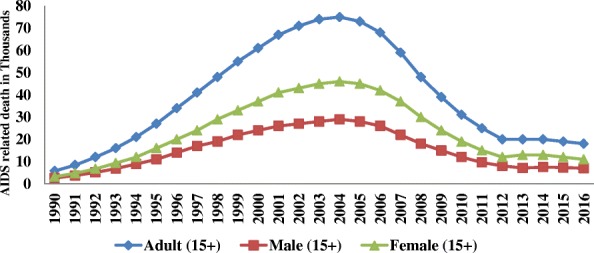
Number of AIDS related death in thousands by sex among adults of Ethiopia, 1990–2016

**Fig. 7:**
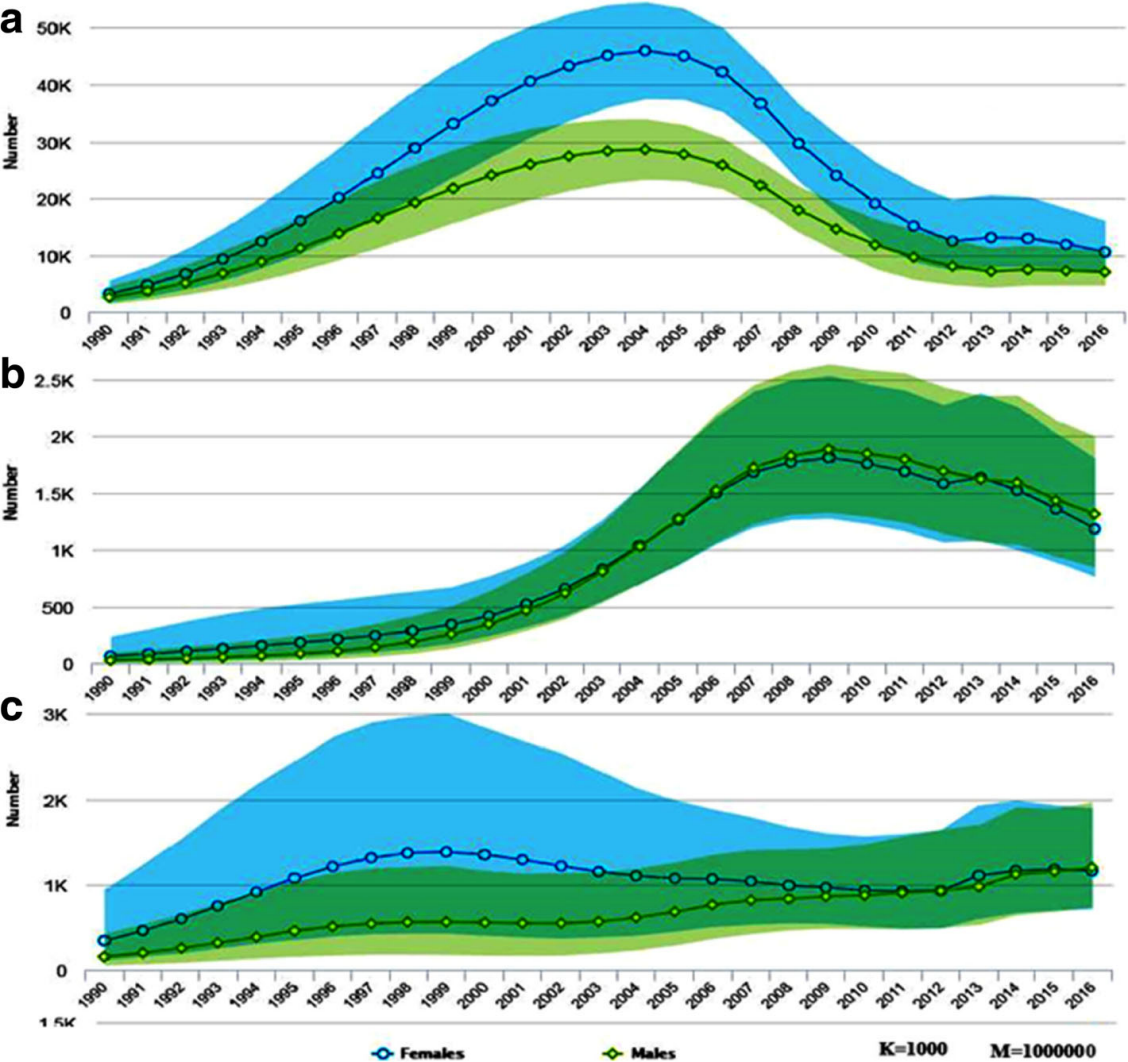
Number of AIDS related death by age and sex among adults (**a**), adolescents (**b**) and young ages (**c**) of Ethiopia, 1990–2016

In 2016, there were 18,000 AIDS related deaths among adults (age 15+), which is 90% of the 20,000 total AIDS related deaths among the general population. Of which 61% (11,000) of deaths were among adult women. Adolescent females (age 10-19) and young women (age 15-24) contributed 48% of the 2500 deaths and 50% of the 2400 deaths in their respective age category. Generally, AIDS-related death in these special age groups accounts for 27% of total AIDS-related deaths estimated among adults of both sex (Figs. 6 and 7).

AIDS-related death among adults reached peak in 2004 with 75,000 deaths. Since then, it gradually declined in subsequent years and reached 18,000 deaths in 2016, which is a 76% reduction from the maximum recorded AIDS-related death in the age group. The number of women dying of AIDS-related illnesses has been declined by 76% in twelve years, from 46,000 in 2004 to 1100 in 2016. The rate of decline for males is also similar; it declined from the peak of 29,000 deaths to 7000 deaths with in the same period. However, the rate of decline in AIDS related mortality among adults since 2012 was steady with only a 1% decline (Fig. 6).

## Discussion

HIV/AIDS has remained one of the greatest challenges of humanity [4, 9]. It is causing devastation in developing countries more apparently in African countries including Ethiopia. Within three decades, since first case of HIV was reported in Ethiopia, almost 1.52 million adults have been infected with HIV and about 1.05 million have died of AIDS-related causes [16–18]. While the epidemic is a potential risk to everyone, some social and age groups are at greater risk of acquiring the virus than others because of their peculiar vulnerability in a particular society [19–24].

The global trend of HIV/AIDS show that the epidemic is affecting more women than men and the number of women living with the virus is greater than that of men by a large margin [2, 4]. To this extent, in many societies, women and girls constitute one of the particularly vulnerable groups for HIV infection [1, 4, 9]. The increased vulnerability in acquisition of the virus boosted with lower access for treatment and care poses a higher risk of mortality among women. Thus the vulnerability of women mainly attributed to socio-economic, cultural and biological factors that increase their exposure and limit access to HIV care [19, 20, 22–24].

Even though, the Ethiopian national HIV prevention and treatment program has made considerable progress in addressing HIV epidemic and averted many more new infection and AIDS related death through early diagnosis, treatment and care, still HIV is a public health problem in the country and the number of individuals accessing care are below expectation [5–7]. Moreover gender disparity observed in the epidemiological trend of HIV infection and treatment is a major concern [6–9].

Besides female are nearly 51% of the national populations, adult females accounted 61.5% of the HIV cases among adults age 15 and above [5, 15–17]. While, adolescent females (age 10-19) and young women (age 15-24) accounted 52.3 and 57.5% of cases among their age category [5, 14]. The overall prevalence rate of HIV/AIDS is 1.62 times higher among adult women than men [14, 15]. Similarly, adult women disproportionately accounted 61.5% of the incidence among all adults, 74% of new infection among adolescents and 68% of new infection recorded among young age people [14, 16–18].

The gender gap observed in Ethiopia is by far higher than the global and regional figures. Globally in 2015, women constituted 51% of all adults living with HIV and young women made up the 60% of their age category [25]. The gap among newly infected cases wer even higher; they constituted 61.5% of the new infection in Ethiopia [15]. While, in the same year globally women made up only 47% of the new infection among adults [15, 25]. Likewise, the proportion of women among the new infected adults were 35, 31, 38, 29, 32 and 22% in the Caribbean, Eastern Europe and Central Asia, Middle East and North Africa, Latin America, Asia-Pacific and Western and Central Europe and North America respectively. While in sub-Saharan Africa and west and central Africa regions, where the larger cause of transmission is through sexual and gender gap is higher the proportion of women are 56 and 64% respectively [25].

In most developing countries like Ethiopia, the sex ratios of prevalent cases and new HIV infections are more disproportionate where women and girls take the lion’s share of new infections [9, 19–22]. The gaps are even more notable among adolescents (aged 10-19) and young women (aged 15-24). In our study 74% of new infections among adolescents and 68% of new infections among young were among adolescent females and women. In line to our report women made up 66% of new infection in young age groups in sub-Sahara Africa. While 58, 46% and only 29% of new infection among young age groups were females in the world, Caribbean and Western and Central Europe and North America respectively [19–25].

Besides women are said to be biologically more vulnerable to acquire the infection due to the fact that infected semen remains in the vaginal canal for a relatively longer period of time, the exposed mucosal surface area is large and vagina is more susceptible to small tears [3, 20, 22]; the small gender gap observed in more developed countries and the higher gap in less developed countries [1, 3, 19–23] could show social, cultural, economic, legal and other factors that adversely affect their capacity to protect themselves from the risk of HIV infection. Therefore, socioeconomic factors are more determining the likely hood of acquiring the infection among women than the biological factor [1, 3, 9–13].

Their relative lack of power over their body and their sexual lives supported and reinforced by their social and economic inequality that make them such a vulnerable group in contracting and living with the virus. With a varying degree of violence against women, including early marriage, sexual abuse, abduction, rape and harmful traditional practices which is rampant in most developing countries and societies, significantly increase their vulnerability to HIV [9–13].

Unlike the global trend of gender disparity where women accounted for only 35% of global HIV cases in the early epidemic period, almost 50% of the global HIV burden by 2005 and 60% in 2009 of worldwide cases, the gender disparity in the trend of HIV infection in Ethiopia was prominent throughout the course of the epidemics [15, 25]. Women carried the highest burden of HIV new infection and total burden in Ethiopia and most developing countries, where their risky factors are prominent. The relatively shorter period of time that it took for females to overtake the gender gap in HIV prevalence and despite decreasing trend of HIV, increasing the gap of women burden, makes the trend more serious [25–27].

The national scale-up of antiretroviral therapy has been highly accelerated to address patients who need HIV care. Nationally, 95% of the 420,000 clients on ART are adults, of whom 62% are adult women [15, 16]. Meanwhile, 59% of all people living with HIV or 88% of people living with HIV who know their status are on treatment. While, 62% of men and 61% of women from all adults living with HIV were on ART in 2016. The coverage of ART treatment has expanded from 34% in 2010 to 61% in 2016 nearly doubled with in six years [16–18].

The estimated ART coverage in Ethiopia is higher than many of the sub-Saharan countries [15]. This could be due to difference in access, retention and difference in implementation of the program. In Ethiopia, access is highly expanded in current few years, retention is higher (90%) and furthermore integration of the ART program with Health Extension package (HEWs) and involvement of Health Development Army to HIV care services highly contributed [28, 29]. However the coverage of HIV care is still below the expected standard of 90% coverage [15].

The gender gap in the treatment program was not as wide as the gap observed in the prevalence [15–17]. The coverage of ART was 62 and 61% among adult women and men respectively. Even a higher coverage was reported from global report of 2015, that women have a higher coverage of ART than men (52% Vs 41%). This may be due to the increasing coverage of PMTCT service (77%) throughout the world which increased the coverage of the service among women [25].

Meanwhile, the coverage of antiretroviral medicines provided to pregnant women living with HIV to prevent transmission to their children rose from 25% (17–32%) to 69% (50–87%) over the same period, which helped to increase the coverage of ART among adult women living with HIV [15–18]. The coverage is lower than countries like South Africa and Uganda which have coverage of above 95%. Furthermore, implementation of test and treat program, strengthening of PMTCT program and achievement of higher retention in ART program resulted in suppression of viral load and averted many more new infections and AIDS related death [4, 6, 30].

The fact that HIV is one of the major causes of morbidity and mortality in Ethiopia was reported from previous studies [6, 7, 29] as it was in many of the developing countries [30]. It is true in many of sub-Saharan countries where access to life saving HAART is low and the risk of HIV progression and related mortality is high [29, 30]. In 2016, there were 18,000 AIDS related death among adults, which is 90% of the total AIDS related death among the general population. Of whom 61% of deaths were among adult women, 48% among adolescent females (age 10-19) and 50% among young women (age 15-24) in their age category [16–18].

AIDS related death among adults declined by 76% since 2004, where the maximum number was recorded at equal rate among men and women [18]. However the rate of decline in AIDS related mortality among adults since 2012 was steady with only a 1% decline [18]. Much of the decline is due to steep reductions in new HIV infections among adults appreciated since 1995, strengthening of PMTCT service and increases in ART coverage [29–31]. Such a trend in reduction of AIDS related mortality was also achieved in the Caribbean, western and central Europe and North America, Asia and the Pacific and western and central Africa regions [30, 31].

Access to ART has a direct impact on an individual’s risk of death, and the country where one lives has a significant impact on death rates and life expectancy [1, 4, 15]. Generally, early access to life-saving ART highly reduces AIDS-related death. Particularly in low-income countries and high HIV burden countries, annual burden of AIDS related death markedly increases related with low access of ART treatment. Besides early access and economic status of the clients also determines the mortality level of individuals related to HIV. In some low income countries, people living with HIV have 10 to 20 times higher death rates than those living in higher income countries [30].

In Ethiopia, higher treatment coverage along with better adherence (retention to 12th month on treatment) allowed patients to have suppressed viral load. In 2012, 90% of peoples receiving ART were retained for 12 months and 51% (41-63%) of all people living with HIV or 86% of people living with HIV on treatment has viral suppression in 2016 [29]. In addition to this since 2010, around 201,000 deaths (28,714 deaths each year) among the general population were averted with ART and 24,200 new infections were also averted through PMTCT [14, 29].

### Limitations

The findings of this study might suffer from the fact that it is retrospective study and based on records; the reliability of the recorded data couldn’t be ascertained and potential bias associated with estimation is there. The determinants of each outcome and the trend was not addressed which may have influence on the result. This data collected in clinical setups may only account those who presented to medical facilities, tested and reported in the surveillance system. And the change in the surveillance system may have an impact on the reliability of the data.

## Conclusion

HIV/AIDS prevalence, new infection and AIDS related death are by far higher among adult women than men. However the coverage of treatment and HIV care is similar by gender. Particularly vulnerable age groups such as young and adolescent females aged 10 to 24 years take the lion’s share of the new infections and prevalent cases, while their share in AIDS related death is 50%. The pattern of change in the trend of HIV/AIDS infection, treatment and death observed in different phase of the epidemics was similar among both genders. After remarkable decline in incidence and prevalence of HIV infection among both genders, it started to increase since 2009 due to suppressed prevention programs. Therefore due attention is needed to avert gender disparity and current increasing incidence of HIV infection among adults. Community based intervention related to female empowerment, behavioral change and HIV risk reduction is needed with a particular emphasis to adolescents and young women. Furthermore, expansion of early diagnosis and treatment service to all infected individuals through clinical and community based campaign with equitable service accesses and coverage is needed.

## Additional file

Additional file 1: Gender disparity in Epidemiological Trend of HIV/AIDS infection and treatment in Ethiopia. (XLSX 15 kb)

## Abbreviation

AIDS: Acquired Immune Deficiency Syndrome
ART: Antiretroviral therapy
HAART: Highly active antiretroviral therapy
HIV: Human immune deficiency virus
PMTCT: Prevention of mother to child transmission of HIV
